# Fellow-Workers and the Roll of Honour

**Published:** 1915-02-06

**Authors:** 


### ROUND THE WORLD.

[The Editor invites Early Information and Contributions Marked "Fellow-Workers."]

### Fellow-Workers and the Roll of Honour.

It was only to be expected that many of the wounded would sooner or later require expert orthopædic treatment, as the extensive damage done to joints and muscles by modern rifle and shell fire is common; and the decision of the War Office to establish a centre for this at Liverpool shows that such need has already arisen. Mr. Robert Jones, who has been placed in charge of the Military Orthopædic Institution, is, of course, a well-known orthopædic surgeon, lecturer on his special subject at Liverpool University, and a member of the staff of the Royal Southern Hospital and the Royal Country Hospital for Children at Liverpool; he is a Ch.M. Liverpool, F.R.C.S. Edin., and honorary F.R.C.S.I.

The Anglo-Serbian Hospital unit, which left for the Near East recently, is strongly representative of the Royal Free Hospital in that three out of the four leading members of its staff are attached to that institution, whilst the house surgeon is Dr. Dorothy Chick. Another lady student has gone out as a dresser, and there will be five lady orderlies, in addition to nine nurses and four male orderlies.

Mr. James Berry, the chief surgeon to the Anglo-Serbian Hospital, is one of our leading authorities on the surgery of the thyroid gland and also a well-known operating surgeon. A student of St. Bartholomew's, where he won various prizes and scholarships, Mr. Berry became an F.R.C.S. Eng. in 1885, and in the same year was awarded double honours in the M.B. examination, as well as a University scholarship and gold medal in surgery at the B.S. Lond. In addition to his work as senior surgeon and lecturer on clinical surgery at the Royal Free Hospital, he is a consulting surgeon to the Alexandra Hospital for Children with Hip Disease, to the New Hospital for Women in the Euston Road, and to the Royal Buckinghamshire Hospital at Aylesbury. Formerly Professor of Pathology at the Royal College of Surgeons, where many years ago he won the Jacksonian prize, Mr. Berry has won the admiration of a wide circle of fellow-workers by his excellent work.

The chief surgeon of this new war hospital will have associated with him his wife, Mrs. Frances May Dickinson Berry, who at home holds the posts of anæsthetist to the Royal Free Hospital and to the Alexandra Hospital for Children with Hip Disease. Mrs. Berry is an M.D., B.S. Lond., and was awarded honours in the examination for the latter degree. A student of the London School of Medicine for Women, she has done a great deal of first-class work, and held numerous important appointments, including those of house surgeon to the Belgrave Hospital for Children, assistant physician and pathologist to the New Hospital for Women, and assistant medical officer to the Education Committee of the London County Council. Whilst in Serbia she will occupy the post of physician and anæsthetist to the institution of which her husband has charge.

Dr. Laurence Christopher Panting, who has also gone out as surgeon to the Anglo-Serbian unit, is an Oxford and Guy's man who has for some time been practising in Truro. He is, indeed, one of the best qualified men in Cornwall, being an M.D., B.Ch. Oxon., F.R.C.S. Eng., M.R.C.P. Lond.; at one time he was demonstrator of chemistry and physics at Guy's Hospital, where, after qualification, he held the posts of house surgeon and gynæcological assistant. Dr. Panting is also a member of the staff of the Royal Cornwall Infirmary at Truro.

The Royal Free Hospital has also sent its radiographer, Mr. Ernest Ulysses Williams, with the medical expedition to Serbia. Mr. Williams is a King's College man who qualified M.R.C.S., L.R.C.P. Lond. in 1903, and after holding numerous resident medical appointments, including those of house physician to the Royal Free Hospital, house surgeon to King's College Hospital, and senior medical officer to the Mount Vernon Hospital for Consumption, devoted his attention to *x*-ray work. In addition to his duties at the Royal Free Hospital, he is also radiographer to the Queen's Hospital for Children at Bethnal Green.

Dr. Frank Gerard Clemow, who has charge of a hospital unit to assist the Montenegrins, is a well-known European resident of Constantinople, where he was until the recent disturbance of normal life physician to the British Embassy and delegate for Great Britain to the International Board of Health. An M.D. Edin. and D.P.H. Camb., he has specially investigated the geography of disease and also published interesting commentaries on the occurrence of cholera in the Near East. Dr. Clemow, who has also studied various Russian health questions and is a member of the Anglo-Russian Literary Society and of the Russian National Health Society, was made a C.M.G. last year.

Major William Herbert Brailey, R.A.M.C. (T.), who has been appointed consulting ophthalmic surgeon for the Indian sick and wounded, is in private life a specialist practising at Hove. Formerly ophthalmic house surgeon at Guy's Hospital, where he did his clinical work after preliminary studies at Cambridge, he had military experience in the South African War, where he served in the C.I.V., and came home with a medal and three clasps. He mobilised with the 2nd Eastern General Hospital at the outbreak of war. Major Brailey, who is an M.D., B.C. Cantab., M.R.C.S. Eng., L.R.C.P. Lond., is also ophthalmic surgeon to the Brighton Deaf and Dumb Institution.

The late Captain Richard Dominic O'Connor, R.A.M.C., one of the medical victims of the great coast struggle, was an Irishman who qualified from St. Bartholomew's Hospital by taking the conjoint diploma in 1907, immediately after which he entered the Army medical service. His father was also a medical man, being the late Mr. F W. O'Connor, F.R.C.S.I., of Limerick.

The late Captain Michael Joseph Lochrin, R.A.M.C., was another Irish medical man who has fallen at the Front. Qualifying in Dublin in 1904 he entered the Army two years later. Just before the war broke out he had been stationed at Aldershot.

The late Captain Rupert Henry Nolan, R.A.M.C., was a University Hospital man who qualified M.R.C.S. Eng., L.R.C.P. Lond. in 1908. After qualification he went to the L C.C. Asylum at Banstead, where he read for the Army Entrance examination, in which he was successful in the following year, being promoted to captain in due course three years later.

